# Neuralgia of the Fifth Pair Cured by Aconite

**Published:** 1880-12

**Authors:** M. Landesberg

**Affiliations:** Philadelphia.


### ARTICLE VI.

### *A Case of Neuralgia of the First Branch of the Fifth Pair, of Six Years Duration, Cured by Duquesnel's Aconitia.*

BY DR. M. LANDESBERG, OF PHILADELPHIA.

Mrs. B., thirty-five years old, had been suffering from trigeminal neuralgia for over six years previous to her coming under my treatment for progressive myopia. The first attack occurred in the summer of 1873, lasting for over sixteen weeks. From this time paroxysms were repeated with unabated violence, at intervals of two to four weeks, usually attacking one side of the face. In some instances the affection kept on for several days or even weeks. All the different kinds of remedies to which the patient recurred were of no avail. They did neither check the morbid process nor break the violence of the paroxysms.

Patient is of nervous temperament. Mental agitation was one of the most prominent causes of the paroxysms.

During my treating her for progressive myopia I had repeated occasion to observe, from the very beginning, the attacts of trigeminal neuralgia. The prodromal symptoms were slight photophobia and the appearance of a small ring of enlarged vessels around the cornea. Then ciliary neuralgia set in, bearing the features of genuine cyclitis. One spot of the upper ciliary region, outside from the superior muscle, became tender to such a degree that the slightest touch produced the most agonizing pain and intense symptoms of irritation. The latter rapidly increasing in the further course of the affection. The upper lid and its surrounding parts became enormously swollen and puffy, and intense chemosis of the conjunctiva of the eyeball developed. Pupil became extremely contracted, yielding but very slightly to atropia. The pain extended over the whole head. The œdematous parts were flushed, moist with perspiration, and showed increased temperature. In rare instances the lower lid and its surrounding parts also showed slight serious infiltration. Intraocular pressure and background of the eye remained normal. Vision was not impaired.

Induced by Dr. E. C. Seguin's paper on the treatment of trigeminal neuralgia, by Duquesnel's Aconitia, I tried this agent on my patient. The form for administering was as follows :—

| | | | |
|---|---|---|---|
| ℞. | Aconitiæ (Duquesnel's), | gr. ¼ | |
| | Glycerinæ, | ʒj | |
| | Alcohol, | ʒj | |
| | Aq. menth. pip., | ℥ij. | M. |

SIG.—One tablespoonful three times a day.

The therapeutical effect was striking. Perfect recovery was obtained after the patient had used two bottles of the prescription.

Eighteen months elapsed, during which period the patient enjoyed perfect health, when her right eye suffered injury by a blow, which caused intraocular morbid alterations. The exterior of the eye remained normal. On the third day after the occurrence the patient complained of tenderness of the eyeball. On examination, I found one spot of the upper ciliary region tender to the touch. I at first considered this tenderness as a consequence only of the lesion. On the succeeding days the pain became intense, photophobia and lachrymation set in, and slight serous infiltration of the conjunctiva of the eyeball developed. There was no doubt that I had to do here with a relapse of trigeminal neuralgia, provoked, likely, either by the blow itself, or by the mental agitation of the patient consequent upon it.

I recurred to aconitia, which checked the paroxysm in its very beginning.—*Med. and Surg. Reporter.*

